# The combination of SLC7A11 inhibitor and oridonin synergistically inhibits cervical cancer cell growth by decreasing the NADPH/NADP^+^ ratio

**DOI:** 10.1016/j.gendis.2024.101265

**Published:** 2024-03-19

**Authors:** Yajie Liu, Pengxing He, Xubin Ma, Yingqi Tian, Yu Zhang, Yang Wang, Yingjie Jia, Hongmin Liu, Ying Liu, Yichao Xu

**Affiliations:** a Key Laboratory of Advanced Drug Preparation Technologies, Ministry of Education, Co-innovation Center of Henan Province for New Drug R&D and Preclinical Safety, School of Pharmaceutical Sciences, Zhengzhou University, Zhengzhou, Henan 450001, China; b The First Affiliated Hospital of Zhengzhou University, Zhengzhou, Henan 450052, China

SLC7A11, as the core component of system x_c_^−^, protects cancer cells from oxidative-stress-induced cell death like ferroptosis by mediating cystine uptake. Recent studies revealed that SLC7A11 is widely overexpressed in various types of cancer, and inhibition of SLC7A11 could inhibit tumor growth.^1^ Consistently, SLC7A11 was overexpressed in cervical cancer, and the expression of SLC7A11 was associated with the severity of cervical cancer (Fig. 1A; Fig. S1A), making SLC7A11 a promising anti-tumor target. However, SLC7A11 inhibitor imidazole ketone erastin (IKE) alone exerts weak antiproliferative effects in cervical cancer cells (Fig. S1D). Oridonin is isolated from the traditional Chinese herbal *Rabdosia rubescens* and exhibits mild anti-cancer activity against multiple types of tumor cells, including cervical cancer cells. However, the low bioavailability and dose-dependent toxicity of oridonin hinder its clinical application.^2^ Here, we first discovered that combination of SLC7A11 inhibitors and oridonin synergistically inhibited cervical cancer cell growth. Then the pharmacological effect and underlying mechanism of the combination of SLC7A11 inhibitors and oridonin were explored.

**Figure 1:**
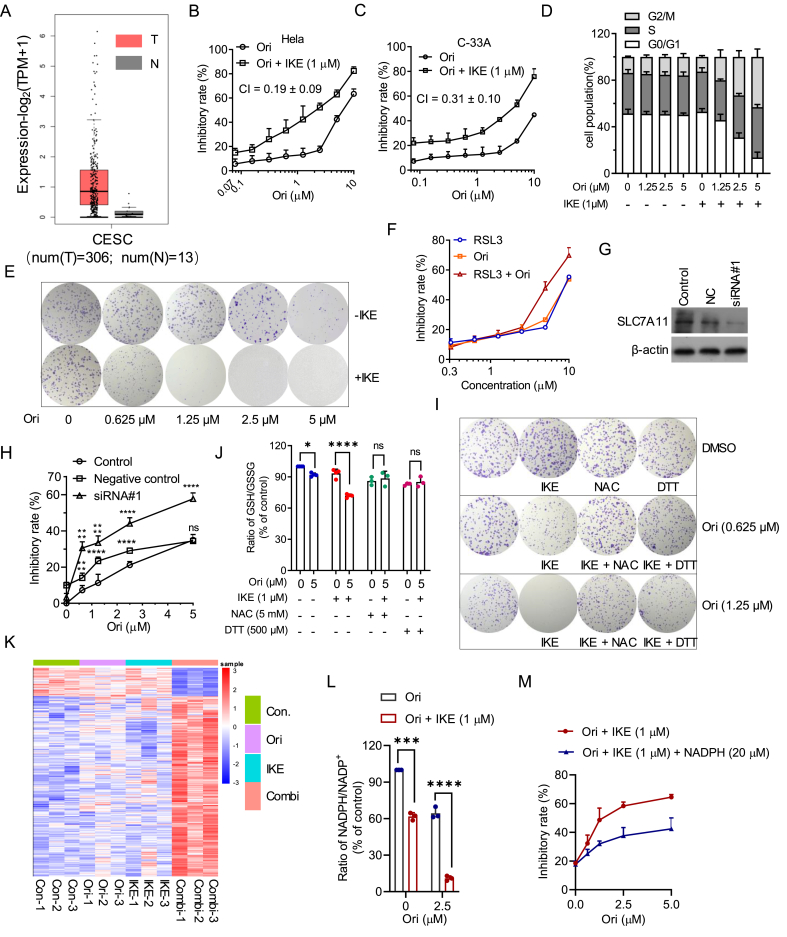
Combination of IKE and oridonin synergistically inhibited cell growth by decreasing NADPH/NADP^+^ ratio. **(A)** The relative transcriptional expression of SLC7A11 in tumor and normal tissues in TCGA-CESC. The data were obtained from GEPIA 2 (http://gepia2.cancer-pku.cn/). **(B, C)** The inhibitory rate of compound was measured by SRB assay treated with oridonin (Ori) alone or together with IKE (1 μM) for 24 h. **(D)** Quantification of cell cycle affected by indicated compounds for 24 h in Hela cells. **(E)** The proliferation was measured by colony formation assay in Hela cells treated with Ori in the absence or presence of IKE (1 μM). **(F)** The inhibitory rate of indicated compounds in Hela cells was measured by SRB assay. **(G)** The protein level of SLC7A11 in Hela cells transfected with scrambled siRNA (NC) or SLC7A11 siRNAs (siRNA#1) for 48 h was detected by western blot. **(H)** The inhibitory rate of oridonin in Hela cells transfected with SLC7A11 siRNAs (siRNA#1) or scrambled siRNA (NC) was measured by SRB assay. **(I)** The proliferation was measured by colony formation assay in Hela cells treated with Ori (5 μM) and IKE (1 μM) in the absence or presence of N-acetyl-l-cysteine (NAC, 5 mM) or DL-Dithiothreitol (DTT, 500 μM). **(J)** Hela cells were treated with indicated compounds for 6 h, and then the intracellular GSH/GSSG ratio was calculated and normalized to the untreated group. Compared with a respective control group using two-way ANOVA. **(K)** Heatmap of genes significantly affected by the combination of Ori (2.5 μM) and IKE (1 μM) for 24 h. The absolute value of foldchange >2. **(L)** Intracellular NADPH/NADP^+^ ratio was calculated and normalized to the untreated group in Hela cells treated with indicated compounds for 6 h. Compared with a respective control group using two-way ANOVA. **(M)** The inhibitory rate of indicated compounds in Hela cells for 24 h was measured by SRB assay. SRB, sulforhodamine B.

Clinical data from TCGA-CESC showed that overexpressed SLC7A11 is associated with the poor overall survival of cervical cancer when the group cutoff was set at the quartile (Cutoff-High: 75%, Cutoff-Low: 25%) instead of the median (Fig. S1B, C), supplying the evidence to target SLC7A11. We previously reported that inhibition of SLC7A11 enhanced the antiproliferative activity of Jaridonin A derivative a2.^3^ Likewise, the combination of IKE and oridonin synergistically inhibited cervical cancer cells with CI values less than 0.5 (Fig. 1B, C; Fig. S2A). SLC7A11 inhibitor sulfasalazine and oridonin also exerted synergistically inhibitory effects against Hela cells, while sorafenib and oridonin showed additive effects (Fig. S2B–D and Table S1). In fact, it has been reported that sorafenib may not be a specific SLC7A11 inhibitor.^4^ Next, the pharmacological investigation showed that the combination of IKE and oridonin synergistically inhibited cell growth and colony formation and induced G2/M phase arrest (Fig. 1D, E; Fig. S2E, F). Notably, cell morphological change induced by the combination of IKE and oridonin was absolutely different from that of oridonin at 40 μM (Fig. S2G). Surprisingly, compounds combination induced apoptosis and mitochondrial membrane potential were mild at 24 h (Fig. S2H–K). Consistently, cleaved caspase 3 and pro-apoptotic protein Bax were slightly elevated by compound combination at 48 h rather than 24 h (Fig. S2J). *In vivo* data showed that the combination of IKE and oridonin suppressed tumor growth more potently than that of oridonin or IKE alone (Fig. S3A). Additionally, the combination of IKE and oridonin had minor effects on the body weight of mice. Collectively, the combination of IKE and oridonin exerted synergistically anti-tumor effects both *in vitro* and *in vivo*.

To explore how the combination of IKE and oridonin synergistically inhibited cell growth, several programmed cell death inhibitors were utilized. Caspase-9 inhibitor Z-LEHD-FMK, pan-caspase inhibitor Z-VAD-FMK, or autophagy inhibitor 3-methyladenine, separately, slightly rescued the cell growth suppressed by the combination of IKE and oridonin at 24 h (Fig. S4A–C). While necrosis inhibitor necrostatin-1 did not affect the antiproliferative effect of IKE and oridonin (Fig. S4D). Given that SLC7A11 negatively regulates ferroptosis, ferroptosis inhibitor ferrostatin 1 was used. Ferrostatin 1 partially rescued the cell growth inhibited by IKE and oridonin (Fig. S4C). Elevation of lipid peroxides and malondialdehyde induced by the combination of IKE and oridonin further confirmed the occurrence of ferroptosis (Fig. S4F–H).

Given that the combination of IKE and oridonin partially induced ferroptosis, we were inspired to test the combination effect of oridonin and GPX4 inhibitor RSL3, which also induced ferroptosis. The combination of RSL3 and oridonin showed additive effects against Hela cells (CI value > 0.8), indicating the importance of SLC7A11 in the combination of IKE and oridonin (Fig. 1F and Table S1). Knockdown of SLC7A11 dramatically elevated the antiproliferative effect of oridonin (Fig. 1G, H). Next, we found that oridonin dose-dependently up-regulated the protein expression rather than the mRNA level of SLC7A11 (Fig. S5A, B). Protein synthesis inhibitor cycloheximide prevented the oridonin-induced SLC7A11 up-regulation (Fig. S5C). Thus, we speculated that oridonin increased SLC7A11 through regulating SLC7A11 translation. Above all, oridonin-induced up-regulation of SLC7A11 is indispensable for the antiproliferative activity of SLC7A11 inhibitors.

Given the important role of SLC7A11 in the combination of IKE and oridonin, and SLC7A11 exerts anti-oxidative activity by supplying cysteine for the synthesis of glutathione (GSH), we determined the ROS content affected by IKE and oridonin. The combination of IKE and oridonin synergistically elevated the ROS level (Fig. S6A, B). Reducing agents N-acetyl-l-cysteine and Dithiothreitol could absolutely eliminate ROS accumulation induced by the combination of IKE and oridonin (Fig. S6C, D). In addition, cell growth and colony formation inhibited by the combination of oridonin and IKE were also largely rescued by N-acetyl-l-cysteine and Dithiothreitol (Fig. S6E; Fig. 1I). Next, we tested the ratio of GSH/GSSG, which plays a major function in ROS homeostasis. Unexpectedly, the combination of oridonin and IKE only weakly reduced the GSH/GSSG ratio (Fig. 1J).

RNA sequencing technique was utilized to explore how the combination of oridonin and SLC7A11 inhibitor induced ROS accumulation (GSE193751). The principal component analysis and heatmap of the top 1351 genes showed that oridonin or IKE alone had minimal effects on transcriptome, while their combination powerfully affected the transcriptome of Hela cells (Fig. S7A; Fig. 1K and Table S2). Given that cysteine can be taken up by system x_c_^−^ or synthesized *de novo* by intracellular trans-sulfuration pathway, we analyzed the transcriptome data and found that the combination of IKE and oridonin significantly elevated the transcription level of cystathionine beta-synthase, a key enzyme in trans-sulfuration pathway (Fig. S7B). In addition, D-3-phosphoglycerate dehydrogenase and phosphoserine aminotransferase, key enzymes for serine *de novo* synthesis, were elevated by the combination of IKE and oridonin (Fig. S7B). Consistently, the combination of IKE and oridonin dramatically elevated the serine content (Fig. S7C). Next, we found that l-serine antagonized the antiproliferative activity of IKE and oridonin (Fig. S7D).

KEGG pathway analysis revealed that the combination of IKE and oridonin affected the biosynthesis of cofactors and folate biosynthesis (Fig. S7E), which are important for nicotinamide adenine dinucleotide phosphate (NADPH) homeostasis.^5^ NADPH, as an essential electron donor, is an important antioxidant by providing reducing equivalents. Then we analyzed genes participating in the consumption and generation of NADPH and found that the combination of IKE and oridonin affected several NADPH-related genes (Fig. S7F). Experimental results showed that the combination of IKE and oridonin dramatically decreased the NADPH/NADP^+^ ratio (Fig. 1L). Additionally, the supplement of NADPH rescued cell growth inhibition and ROS accumulation caused by the combination of IKE and oridonin (Fig. 1M; Fig. S7G, H). Above all, decreased NADPH/NADP^+^ ratio plays an important role in the anti-cancer activity of the combination of IKE and oridonin.

In conclusion, we showed that SLC7A11 inhibitors or low concentration of oridonin alone exerted mild anti-cancer activity, while their combination synergistically inhibited cervical cancer cell growth. Mechanically, we make a hypothesis that oridonin-induced SLC7A11 promotes while the unknown mechanism suppresses cervical cancer cell growth (Fig. S8 Left). The addition of SLC7A11 inhibitor preventes the function of SLC7A11 induced by oridonin (Fig. S8 Right). Importantly, the combination of SLC7A11 inhibitor and oridonin synergistically elevated ROS accumulation by reducing the NADPH/NADP^+^ ratio rather than the GSH/GSSG ratio, which was first discovered by our group. Our study provides the potential drug combination for cervical cancer treatment and also supplies new knowledge for SLC7A11 in cancer cells.

## Author contributions

The work presented was a collaboration of all authors. YCX and YL conceived the project and designed all the experiments. YJL and PXH conducted the majority of experiments and wrote the original draft. XBM, YQT, YZ, YW, KLL, and HML contributed to the analysis of data and drafting of figures.

## Funding

This work was supported by the National Key 10.13039/100006190Research and Development Project (China) (No. 2018YFE0195100), the 10.13039/501100001809National Natural Science Foundation of China (No. 82020108030), and the Key Scientific Research Projects in Colleges and Universities of Henan Province, China (No. 23A350007).

## Conflict of interests

The authors have no conflict of interests to declare.
