# Supplementary material for: The combination of SLC7A11 inhibitor and oridonin synergistically inhibits cervical cancer cell growth by decreasing the NADPH/NADP+ ratio
Source: Genes Dis. 2024 Mar 19;12(1):101265. doi: 10.1016/j.gendis.2024.101265 (PMC11550735; doi:10.1016/j.gendis.2024.101265)
Supplement: Multimedia component 1 [file mmc1.docx]

**Supplementary figures**

**Figure S1. Expression of SLC7A11 is associated with the severity of cervical cancer.**

**Figure S2. Combination of SLC7A11 inhibitor and oridonin synergistically inhibited cervical cancer cell growth.**

**Figure S3. Combination of SLC7A11 inhibitor and oridonin exerted synergistically anti-cancer effects in Hela xenograft mice model.**

**Figure S4. Combination of IKE and oridonin induced multiple programmed cell deaths.**

**Figure S5. Oridonin induced upregulation of SLC7A11 in Hela cells.**

**Figure S6. Combination of IKE and oridonin exerts synergistic anti-cancer activity by causing ROS accumulation.**

**Figure S7. Combination of IKE and oridonin induced ROS accumulation by decreasing NADPH/NADP^+^ ratio in Hela cells.**

**Figure S8. Proposed mechanism by which combination of SLC7A11 inhibitor and oridonin inhibited cervical cancer cells.**

**
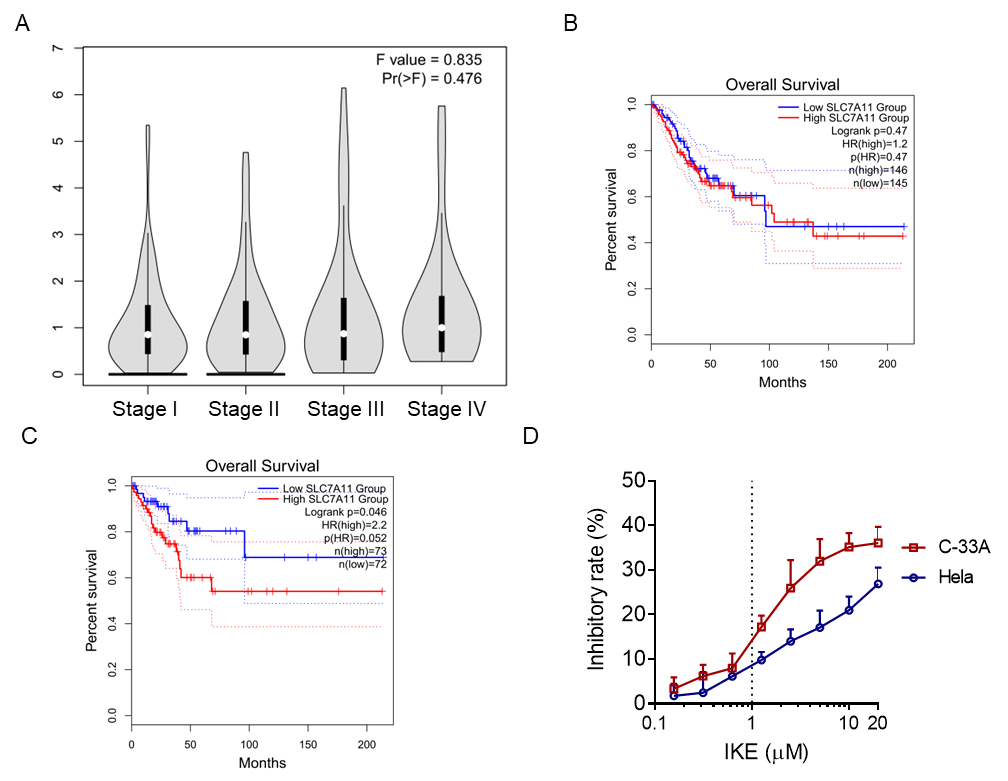
**

**Figure S1. Expression of SLC7A11 is associated with the severity of cervical cancer**. **(A)** The expression of SLC7A11 in 4 cancer stages. **(B)** Correlation between SLC7A11 expression and overall survival of cervical cancer patients, the group cutoff is median. **(C)** Correlation between SLC7A11 expression and overall survival of cervical cancer patients, the group cutoff is quartile. **(D)** The inhibitory rate of IKE was measured by SRB assay in cervical cancer cells treated with diluted concentrations of IKE for 24 h. The data of CESC **(A-C)** were obtained from GEPIA 2 (http://gepia2.cancer-pku.cn/).

**
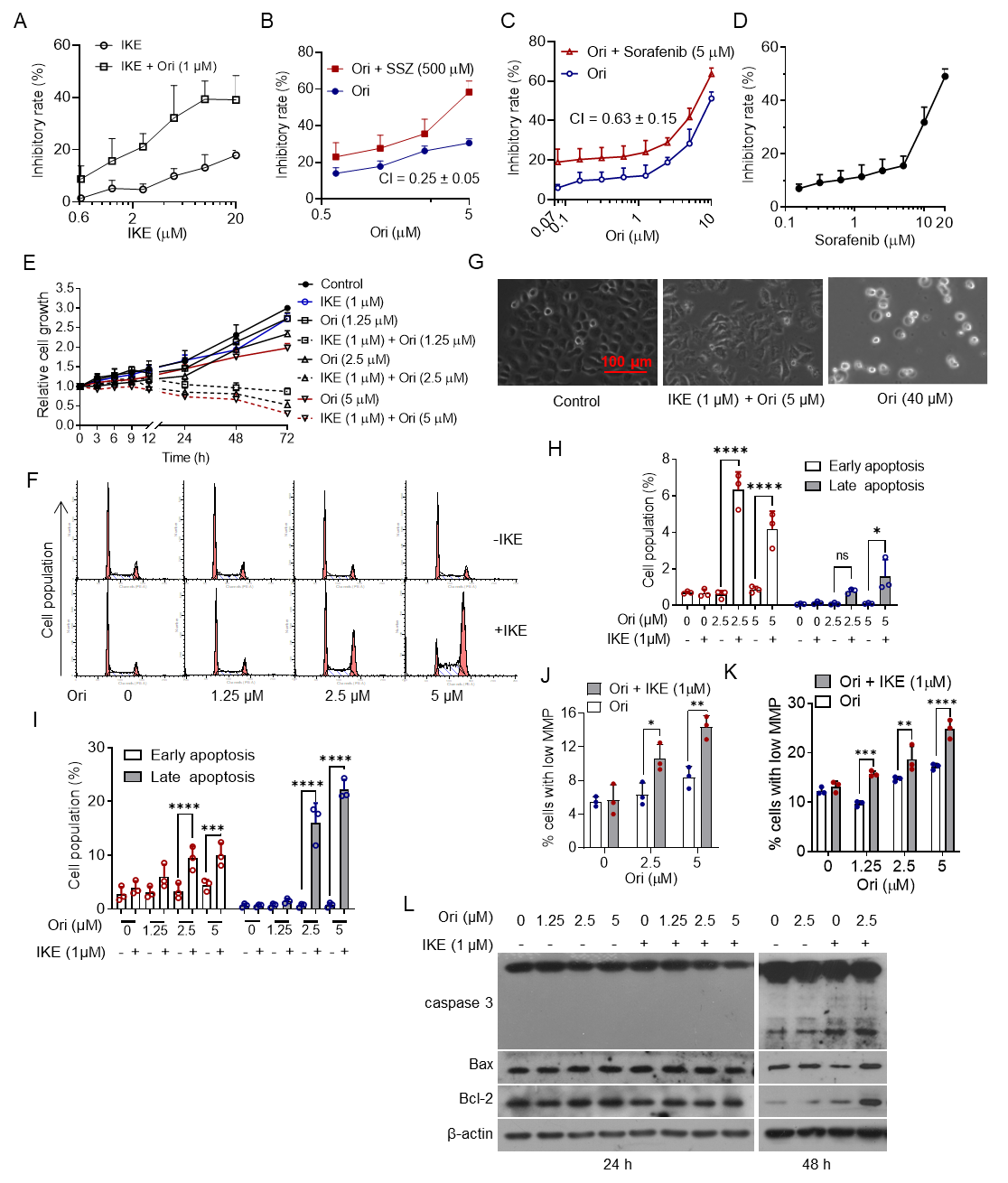
**

**Figure S2. Combination of SLC7A11 inhibitor and oridonin synergistically inhibited cervical cancer cell growth**. **(A)** The inhibitory rate of compound was measured by SRB assay in Hela cells treated with IKE alone or together with oridonin (1 μM) for 24 h. **(B,C)** The inhibitory rate of compound was measured by SRB assay in Hela cells treated with oridonin alone or together with sulfasalazine (SSZ 500 μM) (B) alternatively sorafenib (5 μM) (C) for 24 h. **(D)** The inhibitory rate of compound was measured by SRB assay in Hela cells treated with sorafenib for 24 h. **(E)** Cell growth was detected by SRB assay in Hela cells treated with indicated compounds for different times. **(F)** Cell cycle was tested by flow cytometry in Hela cells treated with oridonin alone or together with IKE (1 μM) for 24 h. **(G)** Hela cells were treated with indicated compounds for 24 h, and then cells were imaged by the bright-field microscopy. **(H,I)** Quantification of Hela cells double-stained by Annexin V-FITC and PI after treatment with indicated compounds for 24 h (H) or 48 h (I), early apoptotic cells mean cells stained by Annexin V-FITC, late apoptotic cells mean cells stained by both Annexin V-FITC and PI. Two-way ANOVA was used to compare the combination group to oridonin treated group. **(J,K)** The ratio of Hela cells stained with low MMP after treated by indicated compounds for 24 h (J) or 48 h (k). The low MMP refers to cell binding to JC-1. Two-way ANOVA was used to compare the combination group to oridonin treated group. (L) The indicated protein level of Hela cells treated with indicated compounds for 24 h or 48 h was detected by western blot.


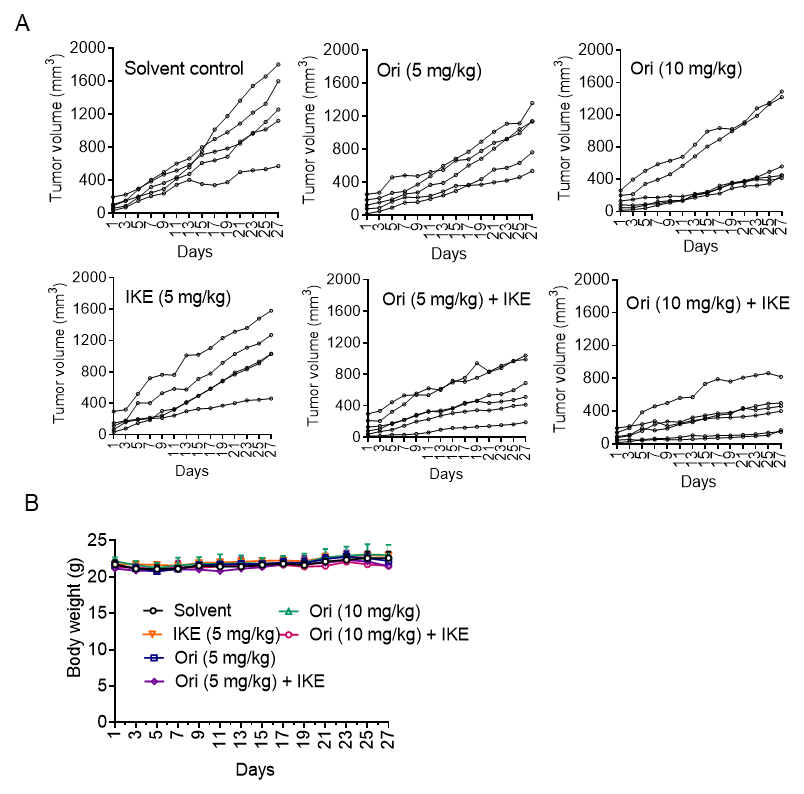


**Figure S3. Combination of SLC7A11 inhibitor and oridonin exerted synergistically anti-cancer effects in Hela xenograft mice model**. **(A,B)** Nude mice bearing tumors were intraperitoneal injected with indicated compounds for 25 days, tumor volumes (A) and mice body weights (B) were measured every two days.


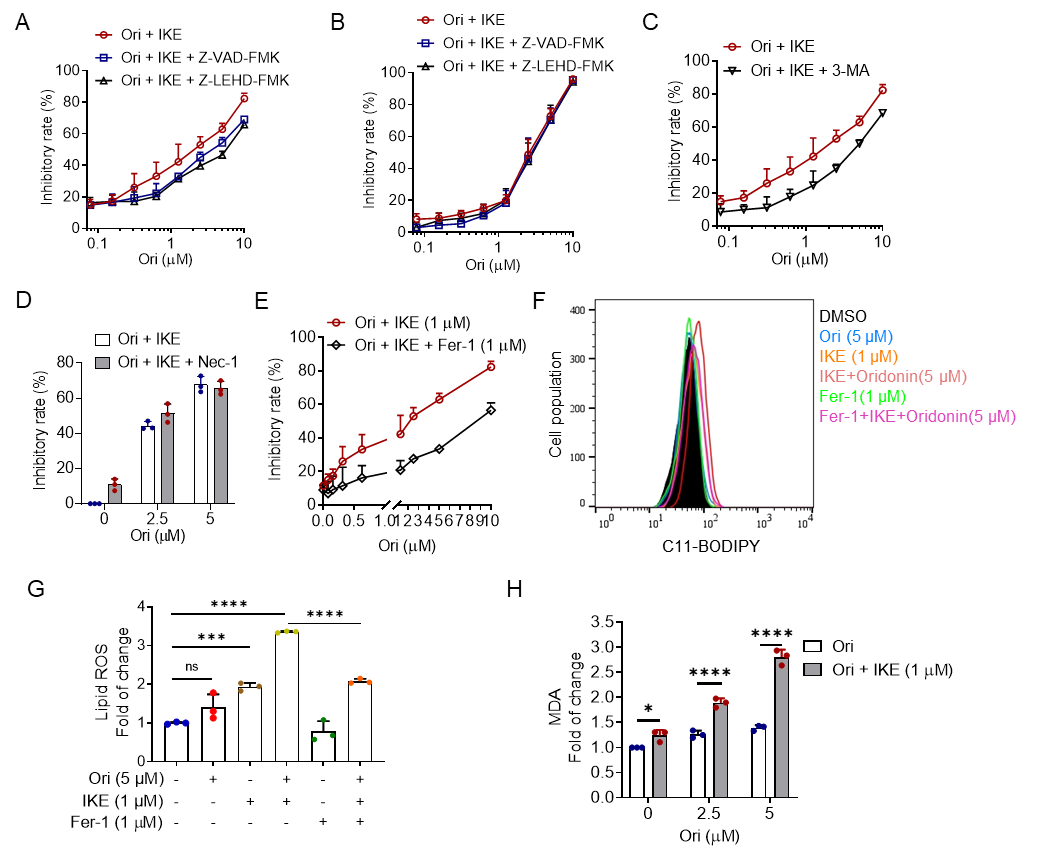


**Figure S4. Combination of IKE and oridonin induced multiple programmed cell deaths**. **(A-D)** Inhibitory rate of indicated compounds was measured by SRB assay in Hela cells treated with indicated agents for 24 h (A, C and D) or 48 h (B). IKE:1 µM, Z-VAD-FMK: 10 µM, Z-LEHD-FMK: 10 µM, 3-MA: 50 µM, Nec-1: 2 µM. **(E)** Inhibitory rate of compounds was measured by SRB assay in Hela cells treated with indicated compounds for 24 h. **(F)** Hela cells were treated with indicated compounds for 6 hours, cells were then stained with C11-BODIPY581/591 and tested by flow cytometry. Concentrations of IKE and Fer-1 were constant. **(G)** Quantification of (F) by FlowJo. Compared with respective control group using two-way ANOVA. **(H)** Intracellular MDA contents were measured in Hela cells incubated with oridonin at the absence or presence of IKE (1 µM) for 6 h. Two-way ANOVA was used to compare the combination group to oridonin treated group.


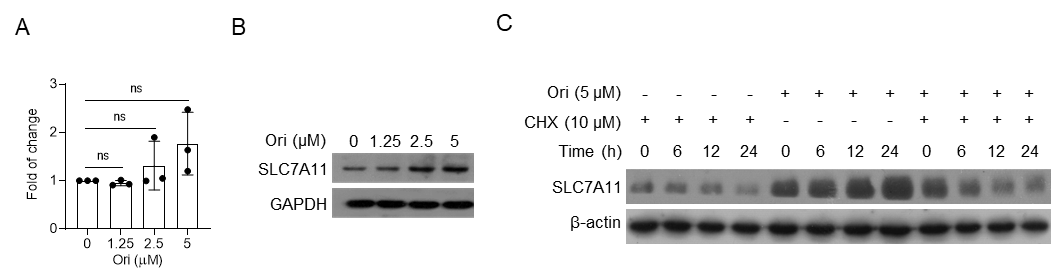


**Figure S5. Oridonin induced upregulation of SLC7A11 in Hela cells**. **(A,B)** The transcription (A) and protein (B) level of SLC7A11 were measured by qRT-PCR and western blot, respectively, in Hela cells treated with oridonin for 24 h. (C) The protein level of SLC7A11 in Hela cells treated with indicated compounds for specific time was detected by western blot.


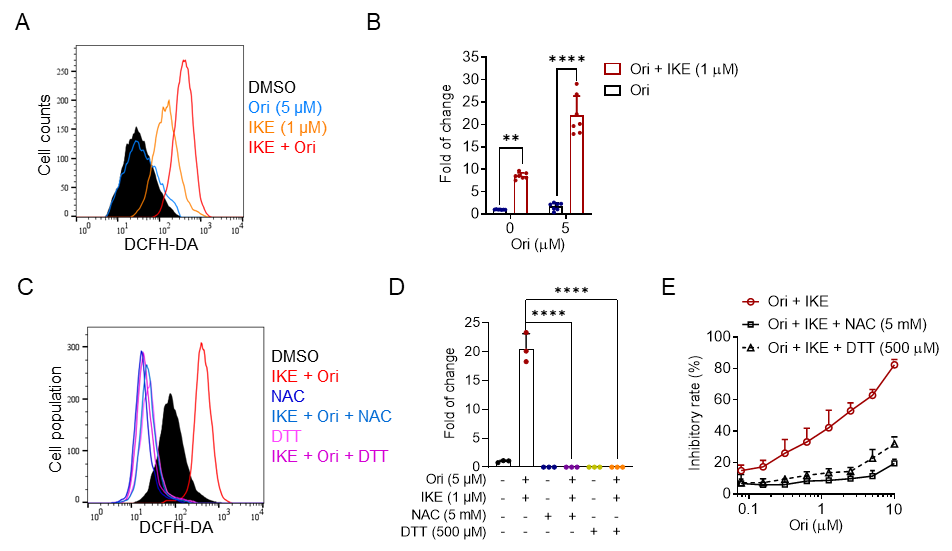


**Figure S6. Combination of IKE and oridonin exerts synergistic anti-cancer activity by causing ROS accumulation**. **(A)** Intracellular ROS was tested by DCFH-DA in Hela cells treated with indicated compounds for 6 h. **(B)** Quantification of (A). Two-way ANOVA was used to compare combination group to single agent group. **(C)** Intracellular ROS was tested by DCFH-DA in Hela cells treated with Oridonin (5 μM) and IKE (1 μM) at the absence or presence of NAC (5 mM) or DTT (500 μM) for 6 h. **(D)** Quantification of (C). Two-way ANOVA was used to compare each group. **(E)** The inhibitory rate of indicated compounds was measured by SRB assay in Hela cells for 24 h, IKE: 1 μM.


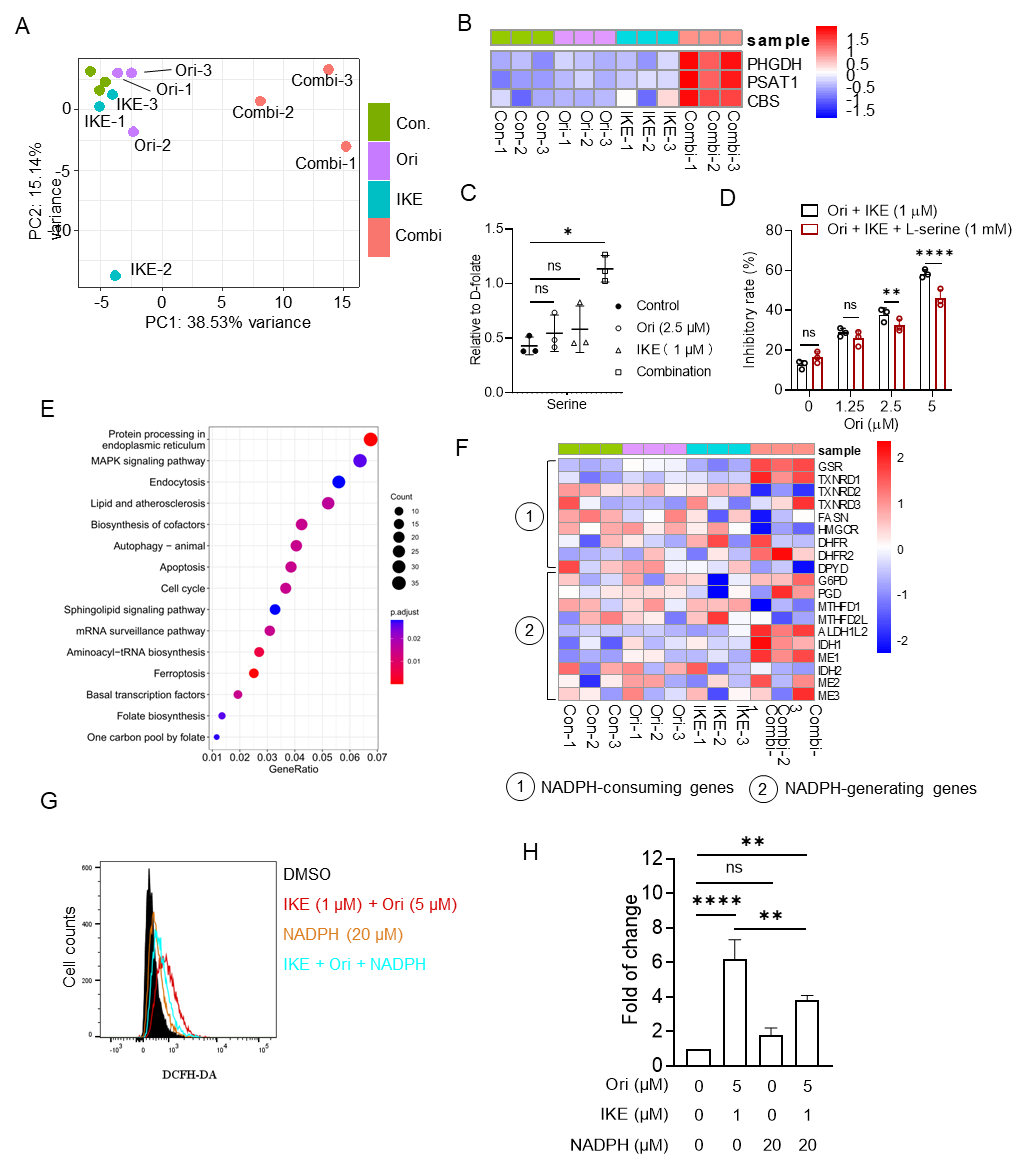


**Figure S7. Combination of IKE and oridonin induced the ROS accumulation by decreasing NADPH/NADP^+^ ratio in Hela cells**. **(A)** Hela cells were treated with Oridonin (2.5 μM), IKE (1 μM) or combination of two compounds for 24 h, cell transcriptomes were then measured and analyzed by PCA. **(B)** Heatmap of indicated genes affected by combination of oridonin (2.5 μM) and IKE (1 μM) for 24 h, absolute value of foldchange > 2. **(C)** Relative content of serine in Hela cells treated with indicated compounds for 24 h. **(D)** Hela cells were treated with indicated compounds for 24 h, inhibitory rate of compound was measured by SRB assay. Compared with respective control group using two-way ANOVA. **(E)** KEGG enrichment analysis was performed to analyze the pathways affected by combination of oridonin (2.5 μM) and IKE (1 μM) for 24 h. **(F)** Heatmap of indicated genes affected by combination of oridonin (2.5 μM) and IKE (1 μM) for 24 h. **(G)** Intracellular ROS was tested by DCFH-DA in Hela cells treated with indicated compounds for 6 h. **(H)** Quantification of (G). Compared with respective control group using two-way ANOVA.

**
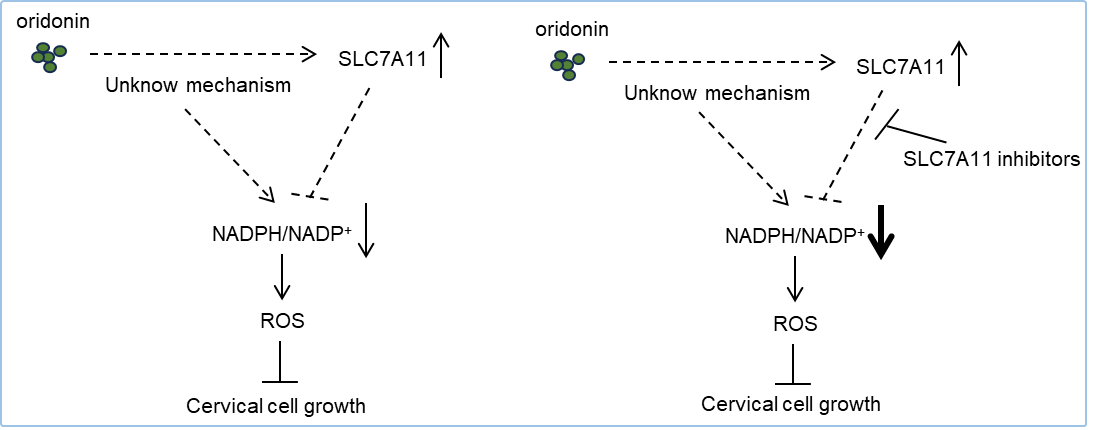
**

**Figure S8. Proposed mechanism by which combination of SLC7A11 inhibitor and oridonin inhibited cervical cancer cells.**

**Material and methods**

Cell lines and cell culture

The human [cervical](javascript:;) [cancer](javascript:;) cell line Hela was obtained from Chinese Academy of Science Cell Bank (Shanghai, China) and had been verified by STR in Shanghai XP Biomed Ltd (Shanghai, China). The human [cervical](javascript:;) [cancer](javascript:;) cell line C-33A was gained from National Infrastructure of Cell Line Resource (Beijing, China). Hela cells were cultured with RPMI 1640 (BI, Israel) containing 10% fetal bovine serum (FBS, BI, Israel). C-33A cells were cultured with DMEM (BI, Israel) containing 10% FBS. All cell lines were cultured in 5% CO2 and 37 °C conditions.

Reagents and antibodies

Z-VAD-FMK, 3-Methyladenine (3-MA), ferrostatin-1 (fer-1), Necrostatin-1 (Nec-1), cycloheximide (CHX) and L-serine were purchased from MedChemExpress (NJ, USA). Z-LEHD-FMK, oridonin, imidazole ketone erastin (IKE), sorafenib and sulfasalazine were purchased from Selleck Chemicals (Houston, USA). DL-Dithiothreitol (DTT), N-acetyl-L-cysteine (NAC), Nicotinamide adenine dinucleotide phosphate (NADPH) and 2’,7’-dichlorodihydroflurescein diacetate (DCFH-DA) were obtained from Beyotime Biotechnology (Shanghai, China).

Antibodies against Caspase 3 (#9662), Bax (#5023) and Bcl-2 (#15071) were purchased from Cell Signaling Technology (Boston, MA, USA). Antibodies against SLC7A11 (ab175186) were obtained from Abcam (Cambridge, UK). Antibody against GAPDH (AB-P-R001) was acquired from Goodhere Biological Technology (Hangzhou, China). Antibody against β-actin (66009-1-lg) was acquired from Proteintech group (Wuhan, China).

SRB assay

Cell viability was measured using the sulforhodamine B (SRB, Beyotime, Shanghai, China) assay, as previously described.^[1]^ Briefly, cells were seeded into 96-well plates and treated with indicated agents for 24 h, then cells were stained by SRB and measured by microplate reader at 560 nm. The inhibitory rate of compound was calculated by the formula: Inhibitory rate (%) = (OD_control_ - OD_treated_) / OD_control_ *100. OD_control_ represents the optic density of cells incubated with DMSO.

**Calculation of Combination Index (CI)**

For the combination of compound A at different concentrations and compound B at constant concentration, CI value was calculated by formula: CI value = IC_50_ (compound A combining with compound B) / IC_50_ (compound A alone), according to previously reported.^[2]^ For the combination of compound A and compound B at constant ratio (A:B), CI value was calculated by CompuSyn software according to Chou-Talalay method^[3]^. Synergistic effect: CI value < 0.8; Antagonistic effect: CI value > 1.2; Additive effect: 0.8 < CI value < 1.2.

Colony formation assay

Hela cells were seeded in 6-well plates at the density of 900 cells per well. Then cells were treated with indicated compounds for 14 days. The cell colonies were fixed with 4% [paraformaldehyde](javascript:;) and stained by crystal violet. Colony numbers were counted by software Image J. The colony formation rate was calculated by the formula: Colony formation rate (%) = Colony number_treated_ / Colony number_control_ *100. Colony number_control_ stands for the colony number of cells incubated with DMSO.

Flow cytometric analyses

Annexin V-FITC/propidium iodide (PI) apoptosis detection kit and mitochondrial membrane potential assay kit were purchased from Beyotime Biotechnology (Shanghai, China). Cell cycle staining kit was obtained from MultiSciences Biotechnology (Hangzhou, China). In strict accordance with manufacturer’s instructions, cell apoptosis, cell cycle and mitochondrial membrane potential (MMP) of cells were measured by flow cytometer. Quantification of data was performed using Flowjo software.

ROS and lipid ROS assay

DCFH-DA (Beyotime, Shanghai, China) and C11-BODIPY^581/591^ (Thermo Fisher Scientific, MA, USA) were used to measure the intracellular ROS and lipid ROS content, respectively. Hela cells were seeded into 6-well plates and treated with indicated compounds for specific time. Then, cells were collected and stained by 10 µM DCFH-DA or 2 µM C11-BODIPY^581/591^ for 30 minutes away from light at 37℃. The cells were then tested by flow cytometer.

**Measurement of GSH/GSSG ratio**

GSH/GSSG ratio was measured by the GSH+GSSG/GSH assay kit (ab239709, Abcam, Cambridge, UK) according to the instruction. The assay is based on the glutathione recycling system involving 5,5′-dithio-bis (2-nitrobenzoic acid) (DTNB) and glutathione reductase. DTNB and GSH react to generate 2-nitro-5-thiobenzoic acid which has a yellow color. Therefore, GSH concentration can be determined by measuring absorbance at 412 nm. The GSSG in cells can be reduced back to GSH by glutathione reductase, then GSH reacts with the DTNB again to produce more 2-nitro-5-thiobenzoic acid. In summary, we test the GSH with DNTB without the glutathione reductase, while test the total glutathione with DNTB and glutathione reductase. The content of glutathione was normalized by sample protein concentrations. The GSH/GSSG ratio was calculated using following formula: GSH/GSSG ratio = (content of reduced glutathione)/ ½ *(content of total glutathione- content of reduced glutathione)

Measurement of NADPH/NADP^+^ ratio

NADPH/NADP^+^ ratio assay kit was purchased from Beyotime Biotechnology (Shanghai, China). Total NADPH include NADPH and NADP^+^, NADP^+^ can be reduced to NADPH by glucose-6-phosphate. NADPH can reduce WST-8 to formazan, which can be detected by colorimetry at 450 nm. The content of NADPH was tested by WST-8, while the total NADPH was detected by WST-8 and glucose-6-phosphate. The content of NADPH was normalized by sample protein concentrations. NADPH/NADP^+^ ratio was calculated using following formula: NADPH/NADP^+^ ratio = (content of NADPH) / (content of total NADPH - content of NADPH).

MDA assay

The content of intracellular malondialdehyde (MDA) was measured using lipid peroxidation MDA assay kit (Beyotime, Shanghai, China) according to the manufacturer’s protocol. The assay is based on the reaction of MDA with thiobarbituric acid. Briefly, Hela cells seeded into 60-mm plates were treated with oridonin with or without IKE for specific time. At the end of treatment, cells were collected and homogenized in assay buffer. Then, the sample were measured by microplate reader at 532 nm.

RNA interference

Cells were seeded in 12-well plates. When the cell confluence reached 30% ~ 50%, the cells were transfected with 40 pmol siRNAs by transfection reagent si-RNA-Mate (GenePharma Co., Shanghai, China) according to manufacturer’s instruction. After 24 h, cells were digested and seeded in 96-well plates to test the anti-proliferative activity of indicated compounds. The effect of siRNAs in cells was tested by western blotting. All siRNAs were purchased from GenePharma Company (Shanghai, China). The sense sequences of targeted genes were as follows: SLC7A11-1: 5′-GGAAGUCUUUGGUCCAUUA-3′; Scramble siRNA: 5’-UUCUCCGAACGUGUCACGUTT-3’.

Quantitative real-time PCR

Total RNAs were extracted from cells using the TRIzol reagent as described previously^[4]^. cDNAs were generated by reverse transcription using HiScriptII Q RT SuperMix (Vazyme, Nanjing, China). qRT-PCR was performed with ChamQ Universal SYBR qPCR Master Mix (Vazyme, Nanjing, China) against specific genes. Relative quantity of gene expression was normalized to GAPDH and analyzed by QuantStudio™6 Flex Real-Time PCR System (Thermo Fisher Scientific, MA, USA). The gene-specific primer pairs, which were synthesized by GENEWIZ (Jiangsu, China), were as follows: GAPDH-F: 5′-GCACCGTCAAGGCTGAGAAC-3′; GAPDH-R: 5′-TGGTGAAGACGCCAGTGGA-3′; SLC7A11-F: 5′-GGTCCATTACCAGCTTTTGTACG-3′; SLC7A11-R: 5′-AATGTAGCGTCCAAATGCCAG-3′.

Cervical cancer cell line-derived xenograft (CDX) studies

All animal experiments were performed in compliance with animal care and use committee guidelines approved by Zhengzhou University (Zhengzhou, China). Female nude mice (ages 6–8 weeks) were purchased from Hunan SJA Laboratory Animal Co. (Changsha, China). Female nude mice were subcutaneously injected with Hela cells at the right flank. Once the tumor volume reached 100–200 mm^3^, mice were randomly divided into 6 groups (5 or 6 mice/group) and intraperitoneally injected with Vehicle, 5 mg/kg oridonin, 10 mg/kg oridonin, 5 mg/kg IKE, 5 mg/kg oridonin with IKE, and 10 mg/kg oridonin with IKE once a day. Oridonin was dissolved in 5% DMSO/95% phosphate buffer solution (PBS) to create a 2 mg/mL solution. IKE was dissolved in 5% DMSO/95% Hank’s Balanced Salt Solution (HBSS), pH 4, to create a 1 mg/mL solution. 5% DMSO/95% HBSS, pH 4, was utilized as vehicle for control group. Terminate study until the biggest tumor volume reach 1500 mm^3^. Tumor volume (tumor length × width[^2^](https://www.ncbi.nlm.nih.gov/pmc/articles/PMC8245858/#bib2)/2) and mice body weight were recorded every two days.

RNA sequencing and bioinformatic analysis

Hela cells seeded in 6-well plate were treated with 0.1% DMSO, 2.5 μM of oridonin, 1 μM of IKE, or combination of oridonin and IKE for 24 h. Total RNA of cells were then extracted by TRIzol reagent (Solarbio Science & Technology Co., Beijing, China). cDNA library preparation, RNA sequencing, quality control and transcriptome proﬁling were performed by Majorbio (Shanghai, China). Briefly, total cDNAs of treated Hela cells were collected and sequenced on Illumina Novaseq 6000, and 150 bp paired-end reads were generated. Raw data of RNA sequencing were aligned and quantified, producing the data of sample read counts. RNA-seq data was then analyzed by DEseq2 to generate the differentially expressed mRNAs affected by combination of oridonin and IKE. KEGG (Kyoto Encyclopedia of Genes and Genomes) enrichment analysis was conducted via the R package ‘clusterProfiler’. The data generated in this study are publicly available in Gene Expression Omnibus (GEO) with accession: GSE193751.

Measurement of L-serine

Hela cells seeded in 60-mm plates were treated with indicated compounds for 24h. Then cells were quenched with liquid nitrogen. Metabolites were extracted by 80% methanol:20% water mixture at dry-ice temperature. The plates were repeatedly froze and thawed in liquid nitrogen three times before the cells were scraped into Eppendorf tube. Cell debris was pelleted by centrifugation at 15000g for 15 min at 4 °C and the supernatant was transferred to a fresh tube. Cell debris was dissolved by 1moL/L NaOH solution as protein content. Supernatants were then concentrated using vacuum centrifugation at 30°C and reconstituted with 100 μL of 50% methanol:50% water mixture. Supernatants were transferred into autosampler vials for analysis after centrifuging at 15000g for 15 min at 4 °C. Metabolites were analyzed with Waters 2695 Separations Module using XBridge BEH Amide Column, (2.1 mm X 100 mm, 5 µm, Ireland). Solvent A was Acetonitrile; solvent B was 0.1% (v/v) Acetic acid. The gradient was: 0 min, 20% B; 3 min, 50% B; 5 min, 50% B; 7min, 20% B; 15 min, 20% B. The injection volume was 5 µl. The column temperature was set to 30 ° C, and the flow rate was 200 µl min^−1^. Mass spectrometry was performed on Waters Quattro micro API triple quadrupole tandem MS using the multiple reaction monitoring (MRM) with an ESI source that was set to positive ion mode. Desolvation gas was nitrogen gas. The instrument parameters were as follows: capillary voltage 3000V, ion source temperature 120°C, desolvation gas temperature 300°C and desolvation gas flow 550 L/h.

Statistical analysis

Data were presented as mean ± SD from at least three independent experiments. Statistical differences were analyzed by one-way or two-way ANOVA with GraphPad Prism Software according to experiments. *P* < 0.05 was defined as statistically significant. P < 0.05 (*); P < 0.01 (**); P < 0.001 (***) and P < 0.0001 (****).

1. Wang S, Ma XB, Yuan XH, Yu B, Xu YC, Liu HM. Discovery of new [1,2,4] Triazolo[1,5-a]Pyrimidine derivatives that Kill gastric cancer cells via the mitochondria pathway. **Eur J Med Chem** **2020**, 203**:** 112630.

2. Li X, Tong LJ, Ding J, Meng LH. Systematic combination screening reveals synergism between rapamycin and sunitinib against human lung cancer. **Cancer Lett** **2014**, 342(1)**:** 159-166.

3. Zhang N, Fu JN, Chou TC. Synergistic combination of microtubule targeting anticancer fludelone with cytoprotective panaxytriol derived from panax ginseng against MX-1 cells in vitro: experimental design and data analysis using the combination index method. **Am J Cancer Res** **2016**, 6(1)**:** 97-104.

4. Rio DC, Ares M, Jr., Hannon GJ, Nilsen TW. Purification of RNA using TRIzol (TRI reagent). **Cold Spring Harb Protoc** **2010**, 2010(6)**:** pdb prot5439.
